# Effects of Folic Acid Supplementation on Oxidative Stress Markers: A Systematic Review and Meta-Analysis of Randomized Controlled Trials

**DOI:** 10.3390/antiox10060871

**Published:** 2021-05-28

**Authors:** Omid Asbaghi, Matin Ghanavati, Damoon Ashtary-Larky, Reza Bagheri, Mahnaz Rezaei Kelishadi, Behzad Nazarian, Michael Nordvall, Alexei Wong, Frédéric Dutheil, Katsuhiko Suzuki, Amirmansour Alavi Naeini

**Affiliations:** 1 Cancer Research Center, Shahid Beheshti University of Medical Sciences, Tehran 14167-53955, Iran; omid.asbaghi@gmail.com; 2 Nutrition and Metabolic Disease Research Center, Department of Nutrition, Faculty of Paramedicine, Ahvaz Jundishapur University of Medical Sciences, Ahvaz 61357-15794, Iran; matina_844@yahoo.com; 3 Nutrition and Metabolic Diseases Research Center, Ahvaz Jundishapur University of Medical Sciences, Ahvaz 61357-15794, Iran; damoon_ashtary@yahoo.com; 4 Department of Exercise Physiology, University of Isfahan, Isfahan 81746-73441, Iran; will.fivb@yahoo.com; 5 Department of Community Nutrition, School of Nutrition and Food Science, Isfahan University of Medical Sciences, Isfahan 81746-73461, Iran; m.rezaei81@yahoo.com; 6 Student Research Committee, Lorestan University of Medical Sciences, Khorramabad 68138-33946, Iran; nazarianbehzad969@yahoo.com; 7 Department of Health and Human Performance, Marymount University, Arlington, VA 22207, USA; michael.nordvall@marymount.edu (M.N.); awong@marymount.edu (A.W.); 8 CNRS, LaPSCo, Physiological and Psychosocial Stress, CHU Clermont-Ferrand, University Hospital of Clermont-Ferrand, Preventive and Occupational Medicine, WittyFit, Université Clermont Auvergne, F-63000 Clermont-Ferrand, France; fred_dutheil@yahoo.fr; 9 Faculty of Sport Sciences, Waseda University, 2-579-15 Mikajima, Tokorozawa 359-1192, Japan

**Keywords:** folic acid, supplementation, folate, oxidative stress, meta-analysis, systematic review

## Abstract

(1) Background: This systematic review and meta-analysis aimed to assess the effects of folic acid supplementation on oxidative stress markers. (2) Methods: Online database including PubMed, Scopus, Web of Science, and Cochrane were searched up to January 2021, to retrieve randomized controlled trials (RCTs) which examined the effect of folic acid supplementation on markers of oxidative stress. Meta-analyses were carried out using a random-effects model. I^2^ index was used to evaluate the heterogeneity of RCTs. (3) Results: Among the initial 2322 studies that were identified from electronic databases search, 13 studies involving 1013 participants were eligible. Pooled effect size from 13 studies indicated that folic acid supplementation elicits a significant rise in serum concentrations of glutathione (GSH) (WMD: 219.01 umol/L, 95% CI 59.30 to 378.71, *p* = 0.007) and total antioxidant capacity (TAC) (WMD: 91.70 umol/L, 95% CI 40.52 to 142.88, *p* < 0.001) but has no effect on serum concentrations of nitric oxide (NO) (WMD: 2.61 umol/L, 95% CI −3.48 to 8.72, *p* = 0.400). In addition, folic acid supplementation significantly reduced serum concentrations of malondialdehyde (MDA) (WMD: −0.13 umol/L, 95% CI −0.24 to −0.02, *p* = 0.020). (4) Conclusions: This meta-analysis study suggests that folic acid supplementation may significantly improve markers within the antioxidative defense system by increasing serum concentrations of GSH and TAC and decreasing serum concentrations of MDA.

## 1. Introduction

Reactive oxygen species (ROS) are highly reactive and toxic molecules produced continuously in the body via the mitochondrial electron transport chain, oxidative metabolism, and immune function [1]. Under normal conditions, ROS are neutralized by a complex antioxidant defense system consisting of enzymatic groups such as catalase, superoxide dismutase (SOD), glutathione reductase (GR), glutathione peroxidase (GPx), and non-enzymatic groups, including reduced glutathione (GSH), carotenoids, ascorbic acid, α-tocopherol, and uric acid [2,3]. Oxidative stress is outlined as an alteration in the pro-oxidant–antioxidant balance in favor of ROS overload that leads to cellular damage [4]. Oxidative stress is established as having a pivotal role in the onset and/or progression of a wide variety of diseases, including cardiovascular disease [5], inflammatory joint disease [6], cancer [7], and diabetes [8]. Oxidative stress has since become a major and contemporary area of scientific interest. Evidence suggests that several dietary factors and strategies have the ability to modulate oxidative stress [9,10,11]. Folate and folic acid, in particular, have received considerable interest in this context and provides the focus of this study [12]. 

Folic acid, known as vitamin B9, is the synthetic form of folate found in fortified food and supplements [13] as it cannot be synthesized by mammals and must be obtained via dietary sources [14]. Folic acid acts as a coenzyme in many important one-carbon metabolic reactions that are necessary for deoxyribonucleic acid (DNA) and ribonucleic acid (RNA) synthesis and various methylation reactions. For example, it is an essential co-substrate in the re-methylation of homocysteine (HCY) to the amino acid methionine [15]. Folic acid has also been reported to have antioxidant, anticancer, cardiovascular, and neuroprotective effects [13]. The antioxidant activity of folic acid is mediated via multiple mechanisms, including a reduction in plasma HCY concentrations, which may increase total antioxidant capacity (TAC) and reduce ROS formation [16]. However, evidence addressing the impact of folic acid supplementation on ROS has been inconsistently portrayed in the literature and varies between populations. For instance, eight weeks of 5 mg per day (mg/d) of folic acid supplementation in women with polycystic ovary syndrome (PCOS) led to decreased malondialdehyde (MDA; an end-product of lipid peroxidation induced by ROS) and increased TAC and GSH concentrations [16]. Contrastingly, Wotherspoon et al. found no significant effect of folic acid administration on biomarkers of oxidative stress in patients with type 1 diabetes [17]. Therefore, we conducted a meta-analysis to obtain a pooled mean effect size estimate of folic acid supplementation on biomarkers of oxidative stress in clinical trials and assess whether folic acid supplementation could ameliorate antioxidant status.

## 2. Materials and Methods

This study was performed based on the Preferred Reporting Items for Systematic Reviews and Meta-Analyses (PRISMA) protocol for reporting systematic reviews and meta-analyses [18].

### 2.1. Search Strategy

To find relevant publications earlier than January 2021, two independent investigators performed a systematic literature search in the following online databases: PubMed, Scopus, Web of Science, and Cochrane. The following terms were used in the search: “folate” OR “folic acid” OR “Vitamin M” OR “Vitamin B9” OR “Folacin” OR “Folvite” OR “Pteroylglutamic Acid” OR “folates” OR “tetrahydrofolates” OR “Formyltetrahydrofolates” AND “Oxidative stress” OR “malondialdehyde” OR “MDA” OR “Glutathione” OR “GSH” OR “Total Antioxidant Capacity” OR “TAC” OR “total antioxidant status” OR “TAS” OR “NO” OR “nitric oxide”. There were no date and language restrictions included in each of the database searches. Furthermore, a manual review of all reference lists from related papers generated from each database search was additionally performed.

### 2.2. Inclusion Criteria

The title and abstract of all identified studies from the database and manual searches were screened for study eligibility. Studies were included in the current meta-analysis if they (1) were randomized controlled trials (RCTs) or placebo-controlled trials, (2) included adult participants ≥18 years old, (3) administered oral folic acid alone (e.g., not in combination with other nutrients), (4) had an intervention duration of at least two weeks, and (5) reported the means and standard deviations (SD) of serum concentrations of NO, MDA, GSH, and TAC at baseline and after the study for both the intervention and control groups.

### 2.3. Excluded Studies

Studies excluded from consideration included (1) a lack of a placebo or control group, (2) interventions utilizing folic acid supplementation as part of a combination of nutrients, (3) in vitro and animal studies, (4) studies incorporating cohort, cross-sectional, and case-control designs, and (5) literature review and/or summary articles.

### 2.4. Data Extraction

Data extraction was conducted independently by two researchers using a standardized data collection form [19]. The following information was extracted from each eligible study: author’s name, year of publication, country of study origin, number, sex, and age of participants, study design, type and dosage of folic acid, baseline (pre) and post-intervention serum concentrations of NO, MDA, GSH, and TAC, and duration of the study. When necessary, data across RCTs reported in dissimilar units were converted to the units presented in this paper in order to perform mean effect size comparisons.

### 2.5. Assessment of Study Quality

We used the Cochrane quality assessment tool to evaluate the risk of bias for each study included in the present meta-analysis. This tool checked seven domains, including random sequence generation, allocation concealment, reporting bias, performance bias, detection bias, attrition bias, and other sources of bias. A domain was assessed a “high risk” score if a study was deemed to have methodological defects that may have distorted results, a “low risk” score if the defect, or lack thereof, was considered ineffectual, and an “unclear risk” score if the information being assessed was not adequate to determine the impact.

### 2.6. Statistical Analysis

Means and standard deviations (SDs) from reported and related data of RCTs were used to estimate the overall effect size on markers of oxidative stress pre to post folic acid intervention. Effect sizes for all variables were expressed as weighted mean differences (WMDs) utilizing 95% confidence intervals (CIs). The random-effects model by DerSimonian and Laird was used to estimate the overall effect sizes [20]. The SDs for mean differences were calculated using the following formula: SD change = square root [(SD baseline) 2 + (SD final) 2 − (2 * R * SD baseline * SD final) [21]. When within-group changes were not reported in RCTs, they were subsequently calculated by subtracting baseline means from final mean values in each distinct group/marker. In studies having only reported standard error of the mean (SEM), SD was obtained using the following formula: SD = SEM * √n, where “n” is the number of participants in each group. Cochrane’s Q test (significance point at *p* < 0.1) and I^2^ index were used to determine the heterogeneity of data between studies [22]. To recognize potential sources of heterogeneity, subgroup analyses were performed. To determine the impression of each study on the pooled effect size, a sensitivity analysis was performed according to established meta-analytic procedures [23]. Moreover, we conducted a one-stage robust error meta-regression (REMR) model which is based on inverse variance weighted least squares regression and cluster robust error variances for the dose-response analysis between magnesium supplementation and glycemic control factors [24]. Finally, funnel plots and Egger’s regression tests were utilized to assess potential publication bias where results from analyses indicated at *p* < 0.05 were considered significantly biased. Statistical analyses were performed with STATA software (version 14.0; StatCorp, College Station, TX, USA). All *p*-values < 0.05 for WMDs were considered statistically significant.

### 2.7. Certainty Assessment

The overall certainty of evidence across the studies was graded according to the guidelines of the GRADE (Grading of Recommendations Assessment, Development, and Evaluation) Working Group. The quality of evidence was classified into four categories, according to the corresponding evaluation criteria: high, moderate, low, and very low [25].

## 3. Results

**Figure 1:**
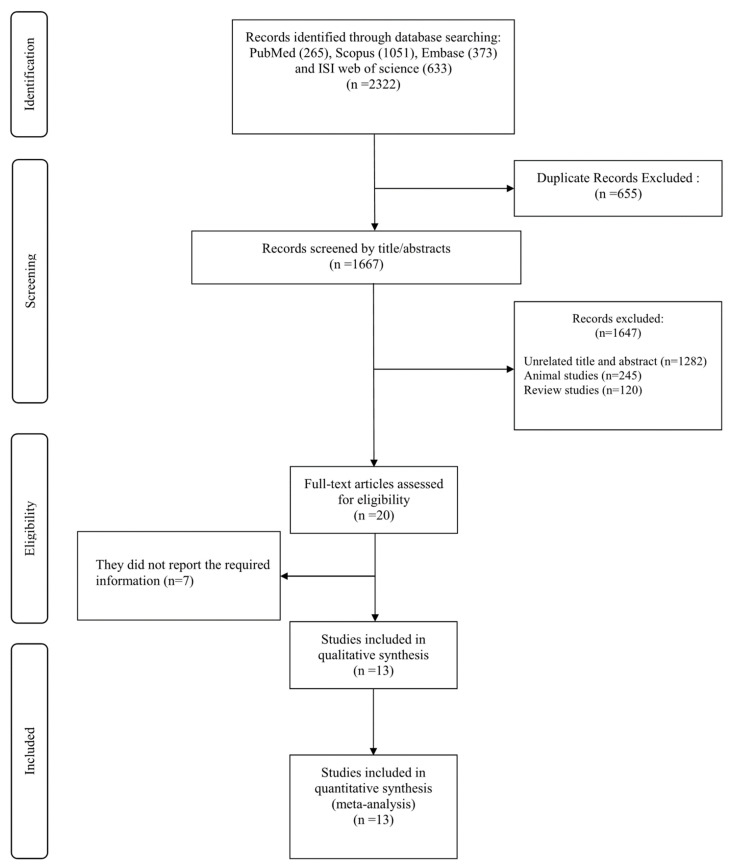
Flowchart of study selection for inclusion studies.

A total of 2322 studies were found from the initial databases and manual references search. From these, 655 duplicates were identified across databases and removed. After screening titles and abstracts for inclusion and exclusion criteria, 1647 studies were excluded for being unrelated or not meeting inclusion criteria (n = 1282), animal studies (n = 245), and review articles (n = 120). Following evaluation of full-text articles from remaining RCTs indicated seven studies did not report required information and thus were excluded from consideration. Finally, 13 studies meeting all inclusion criteria that were not already excluded moved forward for analysis in the current systematic review and dose-response meta-analysis (Figure 1).

### 3.1. Findings from the Systematic Review

The characteristics of the 13 studies included in the present systematic review are shown in Table 1. In total, 1013 adult participants (511 cases and 502 controls), aged ≥18 years, were included in the current meta-analysis study. The countries of origin of RCTs included in the present study were Canada [26], the United Kingdom [27,28,29], Iran [16,29,30,31,32,33,34,35], and the Czech Republic [36]. All included studies incorporated a parallel design that utilized a folic acid dosage between 0.4 and 10 mg/d. Intervention durations across RCTs varied from six to 25 weeks. All selected studies used oral folic acid in various populations, including healthy individuals [37] and in those with pre-existing morbidities and co-morbidities such as coronary artery disease [26,27], high coronary risk [36], hyperlipidemia and hyperhomocysteinemia [29], hypercholesterolemic adults [33], overweight and obese women with polycystic ovary syndrome [16], varicocelectomy [34], cervical intraepithelial neoplasia grade 1 [30], metabolic syndrome [31], endometrial hyperplasia [32], type 2 diabetes [35] and hemodialysis patients [37]. Based on the Cochrane quality assessment tool, the overall quality of 9 RCTs was good, and 4 RCTs were fair (Table 2). 

### 3.2. Findings from the Meta-Analysis

#### 3.2.1. The Effect of Folic Acid Supplementation on Serum Concentrations of NO

**Figure 2:**
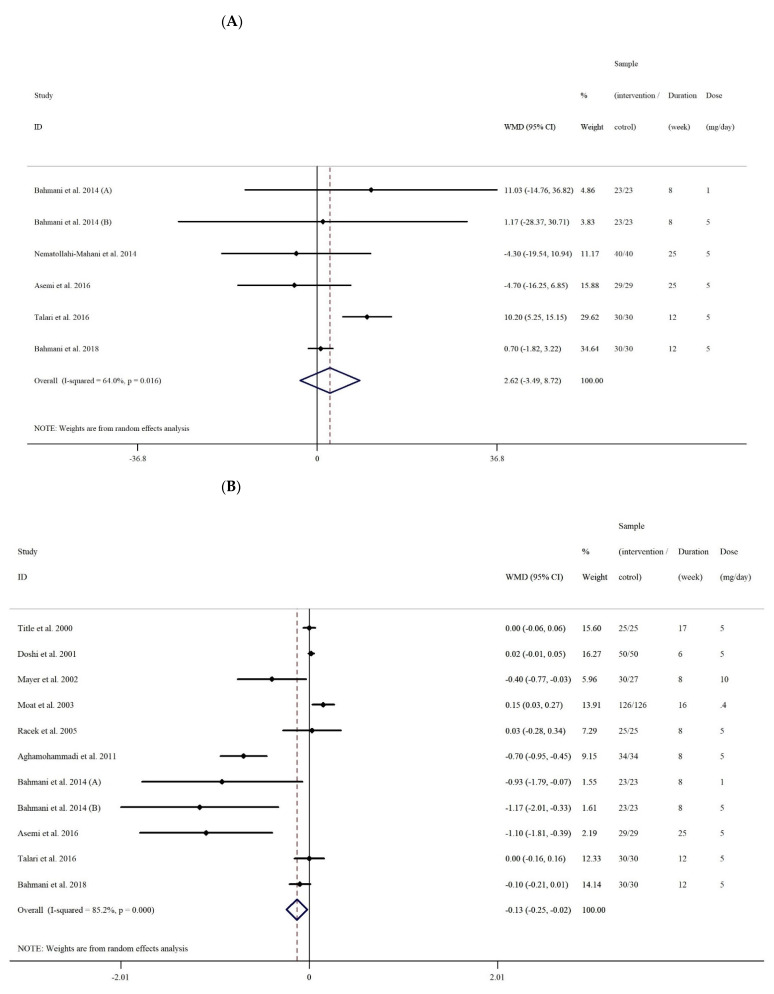

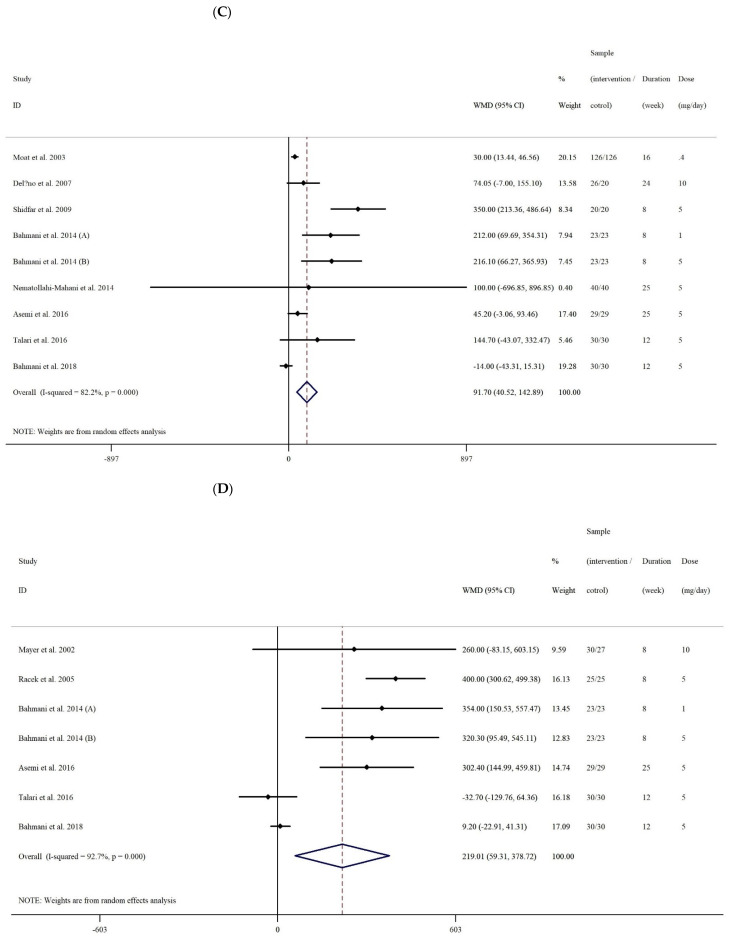
Forest plot detailing weighted mean difference and 95% confidence intervals (CIs) for the effect of folic acid supplementation on (**A**) NO, (**B**) MDA, (**C**) TAC, and (**D**) GSH.

After combining six effect sizes from five studies [16,30,31,32,38], there were no significant lowering effects of folic acid supplementation on serum concentrations of NO when comparing various intervention strategies utilized in participants compared to those in control groups (WMD: 2.61 umol/L, 95% CI −3.48 to 8.72, *p* = 0.400). However, overall between-study heterogeneity was significant (I^2^: 64%, *p* = 0.016) (Figure 2A) and was further detected by subgroup analyses based on intervention duration (≤8 vs. >8 weeks), folic acid dosage (<5 vs. ≥5 mg/d), and participant sex. The results showed that folic acid supplementation had a significant effect on serum concentrations of NO only when the analysis was performed on both sexes (WMD: 10.20 umol/L, 95% CI 5.24 to 15.15, *p* < 0.001) (Table 3).

#### 3.2.2. The Effect of Folic Acid Supplementation on Serum Concentrations of MDA

Ten effect sizes from nine studies were included in this meta-analysis [16,27,30,31,32,35,36,37,38]. Quantitative meta-analysis demonstrated folic acid supplementation had a significant effect on MDA concentrations in interventional participants when compared to their control group counterparts (WMD: −0.13 umol/L, 95% CI −0.24 to −0.02, *p* = 0.020). Significant overall heterogeneity among studies was noted (I^2^ = 75.1%, *p* < 0.001) (Figure 2B). Subgroup analysis was based on intervention duration (≤8 vs. >8 weeks), folic acid dosage (<5 vs. ≥5 mg/d), health status (cardiovascular disease (CVD) and non-CVD), and sex. Results indicated that folic acid supplementation had a significant effect on serum concentrations of MDA when the analysis was performed on non-CVD patients (WMD: −0.35 umol/L, 95% CI −0.60 to −0.01, *p* = 0.005), females (WMD: −0.75 umol/L, 95% CI −1.44 to −0.06, *p* = 0.032), when intervention dose was ≥5 mg/d (WMD: −0.16 umol/L, 95% CI −0.28 to −0.04, *p* = 0.008), and study duration was ≤8 weeks (WMD: −0.41 umol/L, 95% CI −0.78 to −0.05, *p* = 0.026).

#### 3.2.3. The Effect of Folic Acid Supplementation on Serum Concentrations of TAC

Eight effect sizes from eight studies [16,30,31,32,35,36,37,38] presenting data on folic acid supplementation on serum concentrations of TAC were analyzed. Quantitative meta-analysis showed a significant weighted mean effect of folic acid supplementation on serum concentrations of TAC in the intervention group compared with the control group (WMD: 91.70 umol/L, 95% CI 40.52 to 142.88, *p* < 0.001). In addition, a significant between-study heterogeneity (I^2^: 82.2%, *p* < 0.001) was noted (Figure 2C). Further subgroup analyses were performed according to the categories outlined above. Results demonstrated that folic acid supplementation significantly increased serum concentrations of TAC when study duration was ≤8 weeks (WMD: 262.63 umol/L, 95% CI 171.87 to 353.40, *p* < 0.001), the intervention dose was ≥5 (mg/d) (WMD: 113.87 umol/L, 95% CI 30.06 to 197.68, *p* = 0.008), and when the intervention was performed on CVD (WMD: 350 umol/L, 95% CI 213.36 to 486.63, *p* < 0.001) and non-CVD (WMD: 55.01, 95% CI 14.56 to 95.46, *p* = 0.008) participants.

#### 3.2.4. The Effect of Folic Acid Supplementation on Serum Concentrations of GSH

Upon combining seven effects from six studies [16,30,31,32,36,37], a significant difference in serum concentrations of GSH was observed in the intervention compared with the control group (WMD: 219.01 umol/L, 95% CI 59.30 to 378.71, *p* = 0.007) following folic acid supplementation. The heterogeneity among studies was significant (I^2^ = 92.7%, *p* < 0.001) (Figure 2D) and after subgroup analyses, it was found that folic acid supplementation had the effect of significantly increasing serum concentrations of GSH in studies ≤8 weeks (WMD: 374.77 umol/L, 95% CI 294.10 to 455.43, *p* < 0.001), that supplemented with <5 mg/d (WMD: 354 umol/L, 95% CI 150.53 to 557.46, *p* < 0.001) and ≥5 mg/d (WMD: 197.82 umol/L, 95% CI 27.78 to 367.86, *p* = 0.023), that were conducted on CVD (WMD: 400.0 umol/L, 95% CI 300.61 to 499.38, *p* < 0.001) and non-CVD (WMD: 172.01 umol/L, 95% CI 37.03 to 306.99, *p* = 0.012) as well as female (WMD: 233.38 umol/L, 95% CI 14.38 to 452.38, *p* = 0.037) participants.

#### 3.2.5. Publication Bias and Sensitivity Analyses

**Figure 3:**
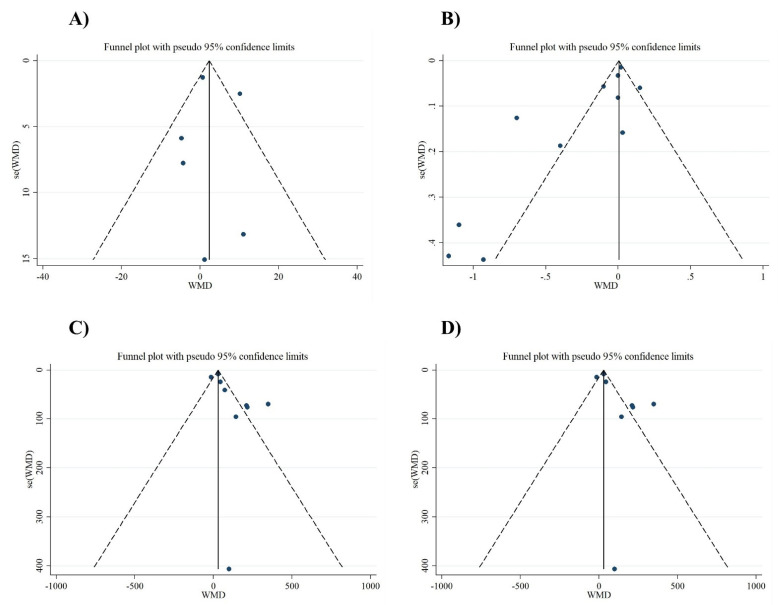
Funnel plot representing publication bias in the studies reporting the effect of folic acid on (**A**) NO (nitric oxide), (**B**) MDA (malondialdehyde), (**C**) TAC (total antioxidant capacity), and (**D**) GSH (reduced glutathione).

Based on visual inspection of funnel plots and Egger’s regression test, we found no evidence of publication bias for NO (*p* = 0.897), MDA (*p* = 0.08), TAC (*p* = 0.05), and GSH (*p* = 0.083) (Figure 3A–D). As such, findings from the sensitivity analyses showed no significant effect of any individual study on the overall effect sizes of serum concentrations of NO, MDA, TAC, and GSH.

### 3.3. Dose-Response Analyses

**Figure 4:**
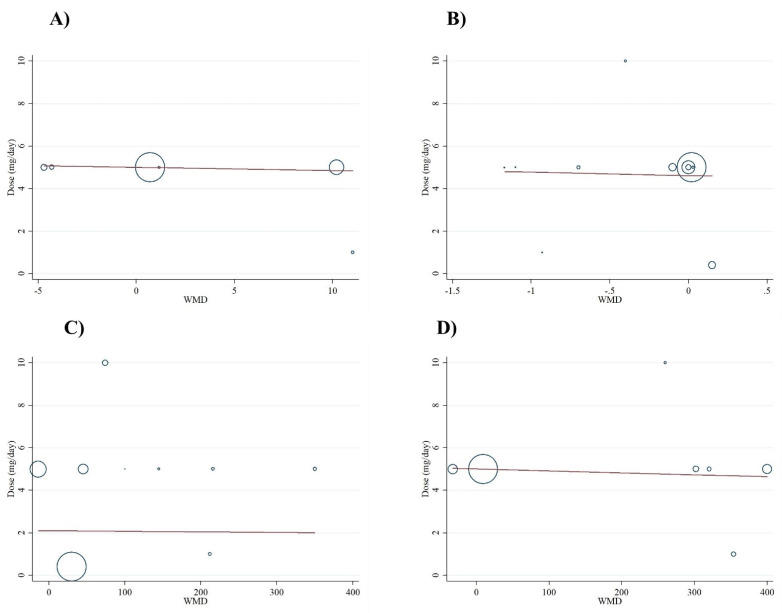
Linear meta-regression plots of the association between dose of folic acid supplementation and weighted mean difference of (**A**) NO (nitric oxide), (**B**) MDA (malondialdehyde), (**C**) TAC (total antioxidant capacity), and (**D**) GSH (reduced glutathione).

**Figure 5:**
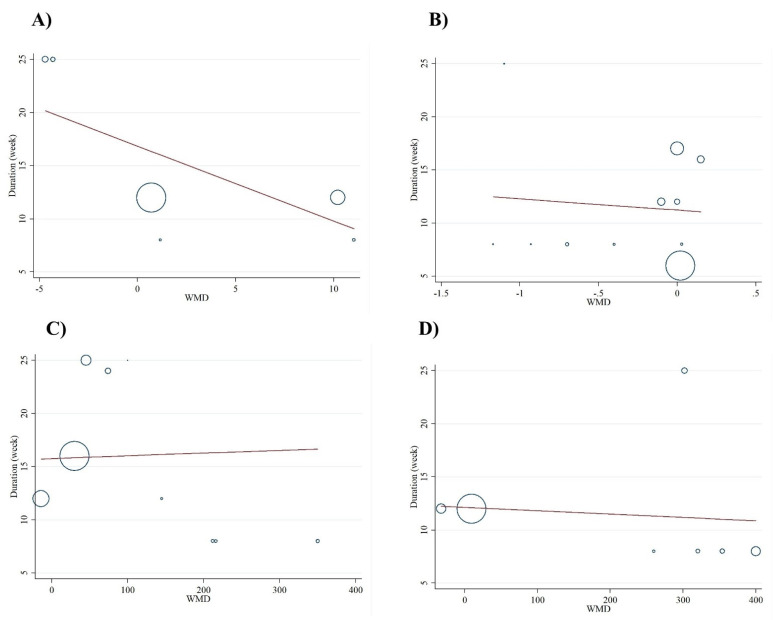
Linear meta-regression plots of the association between duration of folic acid supplementation and weighted mean difference of **A**) NO (nitric oxide), (**B**) MDA (malondialdehyde), (**C**) TAC (total antioxidant capacity), and (**D**) GSH (reduced glutathione).

**Figure 6:**
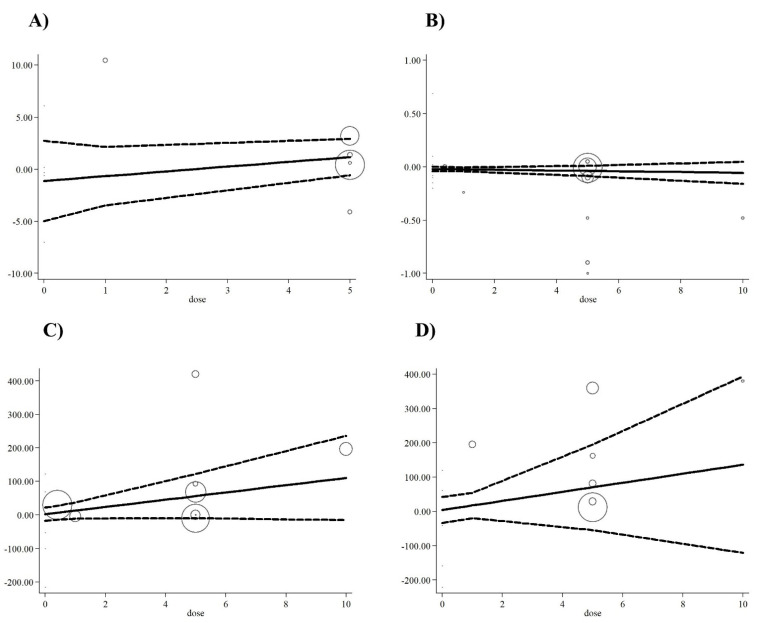
Non-linear dose-response of the association between dose of folic acid supplementation and weighted mean difference of **A**) NO (nitric oxide), (**B**) MDA (malondialdehyde), (**C**) TAC (total antioxidant capacity), and (**D**) GSH (reduced glutathione).

Meta-regression analysis did not indicate a linear relationship between dose (Figure 4A–D) or duration (Figure 5A–D) and serum concentrations of oxidative stress markers (*p* > 0.05). In addition, non-linear dose-response analyses demonstrated the same results for all markers of oxidative stress (Figure 6).

#### Grading of Evidence

The GRADE protocol was used to assess the certainty of the evidence (Table 4). The effect evaluates of NO, MDA, TAC and GSH were regarded as moderate quality. The evidence for NO, MDA, TAC, and GSH was downgraded with low quality for serious heterogeneity and imprecision. The overall quality of the body of evidence of the present systematic review and meta-analysis was regarded as low.

## 4. Discussion

The findings from 13 RCTs included in this meta-analysis indicated that an average folic acid supplementation of 5.1 mg/d (0.4–10 mg/d) with an intervention period lasting between eight to 25 weeks causes a significant rise in serum concentrations of GSH and TAC but has no effect on NO. In addition, a significant reduction in serum concentrations of MDA was noted. Inconsistencies between recent investigations studying the impact of folic acid supplementation on markers of oxidative stress can be attributed to sex differences, variations in dosage, the health status of participants, and/or study duration. For example, subgroup analyses showed that folic acid supplementation among females utilizing an intervention duration of eight weeks or less was associated with significantly increased serum concentrations of GSH; an effect not observed in their male counterparts. In addition, a significant rise in serum concentrations of TAC was only observed in RCTs with an intervention dose of 5 mg/d or more, incorporating a study duration of eight weeks or less. Moreover, females without a history of CVD using an intervention dose of 5 mg/d or more and with a study duration of eight weeks or less showed a significant reduction in serum concentrations of MDA. Nevertheless, similar favorable effects of folic acid supplementation on serum concentrations of GSH and TAC were noted in both healthy and CVD participants in the present study. 

It has been proposed that pro-oxidative status is a critical driver in the pathogenesis and progression of many chronic diseases, including cancer, cardiovascular disease, lung disease, and chronic kidney disease [35]. Low plasma concentrations of TAC may represent an imbalance between the ROS producing and scavenging system (the latter including enzymatic and non-enzymatic antioxidants) [36]. In addition, excessive free radicals may result in reduced plasma concentrations of GSH, a known important mediator of the antioxidant defense system [37]. Due either to its function as a cofactor for glutathione related enzymes or its antioxidant properties in combination with other dietary antioxidants, such as ascorbic acid (vitamin C), α-tocopherol (vitamin E), folate, β-carotene, ubiquinone (coenzyme Q10), bioflavonoids, and selenium, folic acid has beneficial effects to ameliorate oxidative stress status [38,39]. 

Pooled analyses of seven studies included in this meta-analysis found that folic acid supplementation led to a significant increase in serum concentrations of GSH. As noted, the results obtained from subgroup analyses showed a significant rise in serum concentrations of GSH in only females with study durations of eight weeks or less. One possible explanation for such heightened GSH effects of folic acid supplementation in females may be related to the susceptibility of folate depletion or deficiency in this population at the onset of intervention in RCTs. As such, there is no information concerning baseline folate concentrations of participants in the included RCTs; however, in a study conducted by Asemi et al., results of dietary folic acid intakes of participants were found to be lower than the recommended dietary allowance (243.5 versus 400 µgr/day) among women diagnosed with cervical intraepithelial neoplasia at baseline [30]. Whether these results translate to other female populations warrants further investigation. It is also worth mentioning that results from a recent global assessment showed the prevalence of folate deficiency among women of reproductive age to be more than 20 percent in low-income countries, despite the majority of RCTs included in the present meta-analysis coming from developed nations [38]. In addition, female participants in the included studies were more likely to have low serum concentrations of GSH at baseline since higher concentrations of HCY in females would result in depletion of serum GSH concentrations, which in turn can worsen pro-oxidative status [39]. In effect, Bahrami et al. showed that five mg/d folic acid supplementation promoted higher serum concentrations of GSH and TAC among females with homocysteinemia (>15 µmol/L) in a population otherwise susceptible to GSH depletion/deficiency [16]. It seems reasonable to assume that GSH increasing effects of folic acid supplementation in females are due to a degree attributable to a positive relationship between serum concentration of folate and estrogen concentrations, which has been proposed to be a contributory factor to reduce oxidative stress [40]. Consistent with this assumption, two studies have documented estrogen-increasing properties of folic acid supplementation among females [41,42]. 

According to our results, folic acid supplementation involving a study duration of eight weeks or less significantly increased serum concentrations of TAC and GSH and decreased MDA concentrations. The present findings appear consistent with a recent meta-analysis noting that the greatest effect of folic acid supplementation on serum concentrations of C-reactive protein (CRP) was observed in patients following an intervention time period of less than 12 weeks [43]. The lesser effect of folic acid supplementation on serum concentrations of GSH, TAC, and MDA at a longer time of administration is unclear. However, the increasing effects of folic acid supplementation on antioxidant biomarkers with an intervention period lasting eight weeks or less may be attributed to factors influencing bioavailability and bio-efficacy of folic acid supplements that vary between study participants, including gut absorption, nutrient status, food interaction, as well as genetic and host-related factors [44]. Findings from the current study indicated that folic acid supplementation elicits a significant increase in serum concentrations of TAC and GSH among both healthy individuals and CVD participants. Assuming folic acid supplementation improves antioxidant status via a homocysteine-lowering mechanism, it could be concluded that HCY status at the beginning of the study, independent of health status, serves a crucial role in the attributable effects of folic acid supplementation on antioxidative biomarkers. In line with this hypothesis, a study by Racek and colleagues found that folic acid supplementation resulted in increased intraerythrocyte GSH [29] compared to a placebo. Interestingly, the concentrations of HCY were higher than 15 µmol/L (16.9 ± 2.5) in participants, which is considered above the cut-off for homocysteinemia. In another study by Bahmani et al., taking 5 mg/d folic acid supplements for 8 weeks yielded an 11.7 µmol/L increase in serum concentrations of GSH compared to placebo in non-CVD females [16]. Subgroup analysis by dosage in the present study illustrated that the TAC increasing effects of folic acid supplementation remained significant at doses ≥5 mg/d. Shidfar and colleagues similarly showed that the serum concentrations of TAC increased after a daily 5 mg supplementation of folate vs. placebo [33]. Moreover, compared to placebo controls, supplementation of 10 mg folic acid for six months augmented plasma antioxidant capacity in hemodialysis patients [45]. While not directly investigated, such dose-dependent effects of folic acid supplementation on serum concentrations of TAC may not equally translate to lowering HCY since folic acid doses used in all but one study [36] in this meta-analysis have been found to be more than sufficient to reduce HCY concentrations (≥0.8 mg folic acid) [46].

Another important finding of this study was that folic acid supplementation was able to lower serum concentrations of MDA significantly as an overall effect; however, subgroup analyses revealed a significant effect in females. The observed MDA lowering effects of folic acid supplementation in females are in accordance with results from two previous studies conducted in this population [16,30]. When combined with metabolic syndrome where higher concentrations of MDA are observed, such sex-specific lowering effects may be heightened when low-grade inflammation and pro-oxidative status are noted [47]. Accordingly, Bahrami et al. [16] reported a significant rise in plasma concentrations of TAC and a significant reduction in serum concentrations of MDA following folic acid supplementation of 5 mg/d for eight weeks in females with Polycystic ovary syndrome (PCOS), which is a well-established pro-oxidative condition [48]. Further lending evidence to this hypothesis, a study by Aghamohammadi et al. [35] illustrated that pharmacological doses of folic acid supplementation lowered serum concentrations of MDA and increased serum total antioxidant capacity in obese diabetic men. Collectively, it is more likely that the effects of folate therapy on oxidative stress markers are enhanced in individuals prone to or meeting the criteria for metabolic syndrome. From subgroup analysis, it is apparent that folic acid supplementation led to a significant decrease in serum concentrations of MDA among RCTs using an intervention dosage of 5 mg/d or more and a study duration of 8 weeks or less. Consistent with these results, in the study by Racek and colleagues, the combination of 5 mg/d folic acid with antioxidants and folic acid supplementation alone for 8 weeks lowered plasma concentrations of MDA [37]. Our findings show that folic acid supplementation is incapable of exerting an overall influence on serum concentrations of NO although the only included study conducted in both sexes showed increasing effects of folic acid supplementation on NO [31]. However, the results of current studies are insufficient to conclude that folic acid supplementation could alter NO. 

The mechanistic action of folic acid on oxidative stress markers is still not fully understood. The beneficial effects of folic acid supplementation on antioxidant biomarkers could be explained partly through lowered HCY concentrations and/or direct antioxidative effects [49]. Since the earliest meta-analysis confirmed the role of homocysteinemia in the pathogenesis of vascular dysfunction [50], subsequent investigations have studied the effect of folate on reducing plasma concentrations of HCY [51,52,53,54,55]. It is generally accepted that HCY acts as a pro-oxidative agent through the generation of ROS via activation of protease-activated receptors (PARs) and inhibition of endothelial NOS activation [56]. In view of the fact that MDA is an end product of non-specific lipid peroxidation induced by ROS, it can be deduced that hyperhomocysteinaemia gives rise to the higher plasma MDA concentrations via the formation of metabolites and the generation of ROS [57]. While the beneficial effects of folate on concentrations of HCY have received considerable attention, other mechanisms such as modification of transcriptional regulation of nicotinamide adenine dinucleotide phosphate (NADPH) oxidase by folic acid supplementation may partly explain its direct effects on oxidative stress [58]. In addition, a hypothesis originally proposed by Joshhi and colleagues suggested that free radical scavenging properties resulting from folic acid supplementation may be explained by beneficial oxidation of folic acid and repair of thiyl radicals [59]. The exact mechanism responsible for increments of NO is not well understood. However, an increase in NO bioavailability within vascular endothelium following folic acid supplementation has been proposed [60].

The present study comes with notable strengths. To our knowledge, this is the first systematic review and meta-analysis to investigate the effects of folic acid supplementation on a range of biomarkers of oxidative stress, having followed a systematic and internationally recognized consensus methodology. However, the high inter-study heterogeneity with certain biomarkers presents limitations in the interpretability of data. Of the included RCTs, interventions were performed on participants with various baseline medical conditions such as health status, CVD, cervical intraepithelial neoplasia, PCOS, varicocelectomy, metabolic syndrome, hyperlipidemia, and hyperhomocysteinemia, making generalizability of effects difficult at this juncture in time. Moreover, included studies did not report dietary intake of folic acid and/or dietary intake of fortified foods with folic acid. As noted, baseline measurement of folic acid concentrations or dietary assessment of folic acid/folate ingestion via a validated tool such as 24-h food recalls were not analyzed or undertaken in the included RCTs. Future meta-analytic studies on this topic should incorporate the risk of bias assessment of RCTs in order to evaluate the strength of evidence and effect sizes.

## 5. Conclusions

This meta-analysis of RCTs suggested that folic acid supplementation significantly increases serum concentrations of GSH and TAC and decreases serum concentrations of MDA. The greatest impacts were observed in females when the intervention period was eight weeks or less, but caution was warranted when interpreting these results due to a dearth of RCTs. However, well-designed studies for both sexes should continue to be performed to identify optimal dosing and duration effects of folic acid supplementation in targeted populations. 

## Figures and Tables

**Table 1:** Characteristics of included studies.

Study Design	Participant	Sample Size and Sex	Sample Size		Trial Duration (Week)	Means Age		Means BMI			Intervention
			IG	CG		IG	CG	IG	CG	Folic Acid Dose (mg/d)	Control Group
RA/DB/PC (parallel)	Coronary Artery Disease	50: 40M, 10F	25	25	17	57.2 ± 9.8	60.6 ± 8.6	NR	NR	5	Placebo
RA/PC (parallel)	Coronary Artery Disease	50: 44M, 6F	50	50	6	57 ± 8	57 ± 8	28.5 ± 4.4	28.5 ± 4.4	5	Placebo
RA/PC (parallel)	high coronary risk	57: 30M,27F	30	27	8	61.2 ± 24.46	61.2 ± 24.46	28.2 ± 16.53	28.2 ± 16.53	10	Placebo
RA/DB/PC (parallel)	healthy individuals	126 M/F	126	126	16	18-65	18-65	NR	NR	0.4	Placebo
RA (parallel)	Hyperlipidemia and Hyperhomocysteinemia	50: 37M, 13F	25	25	8	59 ± 9.75	56.4 ± 9.75	NR	NR	5	No intervention
RA/DB/PC (parallel)	Hemodialysis patients	46: NR	26	20	24	51.6 ± 10.7	52.3 ± 15	NR	NR	10	Placebo
RA/DB/PC (parallel)	Hypercholesterolemic Adults	40: 16M, 24F	20	20	8	44 ± 7.06	45 ± 7.78	27.06 ± 2.64	26.05 ± 2.17	5	Placebo
RA/DB/PC (parallel)	Type 2 diabetes	68 M/F	34	34	8	58.72 ± 6 7.2	55.6 ± 6 9.3	27.4 ± 6 3.2	27.8 ± 6 4	5	Placebo
RA/DB/PC (parallel)	overweight and obese women with polycystic ovary syndrome	46: 46F	23	23	8	24.1 ± 5.4	24.9 ± 5.9	26.1 ± 6.2	27.6 ± 5.7	1	Placebo
RA/DB/PC (parallel)	overweight and obese women with polycystic ovary syndrome	46: 46F	23	23	8	25.1 ± 4.9	24.9 ± 5.9	29 ± 5.9	27.6 ± 5.7	5	Placebo
RA/PC (parallel)	varicocelectomy	80: 80M	40	40	25	NR	NR	NR	NR	5	Placebo
RA/DB/PC (parallel)	cervical intraepithelial neoplasia grade 1	58: 58F	29	29	25	36.8 ± 8.8	39.1 ± 9.1	28.2 ± 3.5	29.8 ± 6.4	5	Placebo
RA/DB/PC (parallel)	Metabolic Syndrome	60: 26M, 34F	30	30	12	62.1 ± 9.6	65.4 ± 11.5	29.8 ± 3.8	29.8 ± 4.4	5	Placebo
RA/DB/PC (parallel)	Endometrial Hyperplasia	60: 60F	30	30	12	44.4 ± 6.5	44.7 ± 3.1	30.7 ± 4.6	30.5 ± 3.8	5	Placebo

**Abbreviations:** IG—intervention group; CG—control group, DB—double-blinded; SB—single-blinded, PC- placebo-controlled, CO—controlled; RA—randomized, NR—non reported, F—female, M—male.

**Table 2:** Quality assessment.

Studies	Random Sequence Generation	Allocation Concealment	Selective Reporting	Other Sources of Bias	Blinding (Participants and Personnel)	Blinding (Outcome Assessment)	Incomplete Outcome Data	Overall Quality
Title et al., 2000	L	H	H	H	L	H	L	Good
Doshi et al., 2001	L	H	H	H	H	H	L	Fair
Mayer et al., 2002	L	H	H	H	H	H	L	Fair
Moat et al., 2003	L	H	H	H	L	H	L	Good
Racek et al., 2005	L	H	H	H	H	H	L	Fair
Delfino et al., 2007	L	L	L	H	L	L	H	Good
Shidfar et al., 2009	L	H	H	H	L	H	L	Good
Agha mohammadi et al., 2001	L	U	H	H	L	U	L	Good
Bahmani et al., 2014	L	H	L	H	L	H	L	Good
Nematollahi-Mahani et al., 2014	L	H	H	H	H	H	L	Fair
Asemi et al., 2016	L	H	L	H	L	H	L	Good
Talari et al., 2016	L	H	L	H	L	H	L	Good
Bahmani et al., 2018	L	H	L	H	L	H	L	Good

**Abbreviations:** L, low; H, high; U, unclear.

**Table 3:** Subgroup analyses of folic acid supplementation on oxidative stress in adults.

	Number of Effect Sizes	WMD (95%CI)	P within Group	Heterogeneity		
				P Heterogeneity	I^2^	P between Sub-Groups
Subgroup analyses of folic acid supplementation on NO						
Overall effect	6	2.61 (−3.48, 8.72)	0.400	0.016	64.0%	
Trial duration (week)						
≤8	2	6.76 (−12.66, 26.19)	0.495	0.622	0.0%	0.650
>8	4	2.06 (−4.83, 8.96)	0.557	0.004	77.7%	
Intervention dose (mg/d)						
<5	1	11.03 (−14.75, 36.81)	0.402	-	-	0.506
≥5	5	2.12 (−4.33, 8.57)	0.520	0.009	70.3%	
Sex						
Both sexes	1	10.20 (5.24, 15.15)	**<0.001**	-	-	0.002
Female	4	0.55 (−1.88, 2.99)	0.656	0.695	0.0%	
Male	1	−4.30 (−19.53, 10.93)	0.580	-	-	
Subgroup analyses of folic acid supplementation on MDA						
Overall effect	11	−0.13 (−0.24, −0.02)	**0.020**	<0.001	85.2%	
Trial duration (week)						
≤8	6	−0.41 (−0.78, −0.05)	**0.026**	<0.001	89.8%	0.902
>8	5	−0.02 (−0.14, 0.10)	0.747	0.001	78.4%	
Intervention dose (mg/d)						
<5	2	−0.30 (−1.34, 0.74)	0.569	0.014	83.3%	0.031
≥5	9	−0.16 (−0.28, −0.04)	**0.008**	<0.001	86.0%	
Health status						
CVD	3	0.01 (−0.01, 0.04)	0.225	0.857	0.0%	0.012
non-CVD	8	−0.35 (−0.60, −0.10)	**0.005**	<0.001	88.5%	
Sex						
Both sexes	6	0.06 (−0.18, 0.04)	0.239	<0.001	85.9%	0.003
Female	4	−0.75 (−1.44, −0.06)	**0.032**	0.001	81.9%	
Subgroup analyses of folic acid supplementation on TAC						
Overall effect	9	91.70 (40.52, 142.88)	**<0.001**	<0.001	82.2%	
Trial duration (week)						
≤8	3	262.63 (171.87, 353.40)	**<0.001**	0.297	17.6%	<0.001
>8	6	27.90 (−2.72, 57.35)	0.075	0.056	53.7%	
Intervention dose (mg/d)						
<5	2	106.71 (−69.43, 282.85)	0.235	0.013	83.9%	0.612
≥5	7	113.87 (30.06, 197.68)	**0.008**	<0.001	84.4%	
Health status						
CVD	1	350.00 (213.36, 486.63)	**<0.001**	-	-	<0.001
non-CVD	8	55.01 (14.56, 95.46)	**0.008**	0.001	70.4%	
Sex						
Both sexes	4	134.81 (15.51, 254.11)	0.027	<0.001	86.9%	0.344
Female	4	84.36 (−2.07, 170.80)	0.056	<0.001	84.6%	
Male	1	100.00 (−696.84, 896.84)	0.806	-	-	
Subgroup analyses of folic acid supplementation on GSH						
Overall effect	7	219.01 (59.30, 378.71)	**0.007**	<0.001	92.7%	
Trial duration (week)						
≤8	4	374.77 (294.10, 455.43)	**<0.001**	0.815	0.0%	<0.001
>8	3	72.32 (−63.49, 208.13)	0.297	0.001	85.6%	
Intervention dose (mg/d)						
<5	1	354.00 (150.53, 557.46)	**0.001**	-	-	0.004
≥5	6	197.82 (27.78, 367.86)	**0.023**	<0.001	93.2%	
Health status						
CVD	1	400.00 (300.61, 499.38)	**<0.001**	-	-	<0.001
non-CVD	6	172.01 (37.03, 306.99)	**0.012**	<0.001	84.7%	
Sex						
Both sexes	3	204.25 (−134.93, 543.45)	0.238	<0.001	94.7%	<0.001

**Abbreviation:** CI—confidence interval, WMD—weighted mean differences, NO—nitric oxide, MDA—malondialdehyde, TAC—total antioxidant capacity, GSH—glutathione3.

**Table 4:** GRADE profile of folic acid supplementation for NO, MDA, TAC, and GSH scores in adult population.

Quality Assessment						Summary of Findings	
Outcomes	Risk of Bias	Inconsistency	Indirectness	Imprecision	Publication Bias	Number of Intervention/Control	WMD (95%CI)
NO	No serious limitations	serious limitations ^a^	No serious limitations	Serious Limitations ^e^	No serious limitations	175/175	2.61 (−3.48, 8.72)
MDA	No serious limitations	Very serious ^b^	No serious limitations	No serious limitations	No serious limitations	425/422	−0.13 (−0.24, −0.02)
TAC	No serious limitations	Very serious ^c^	No serious limitations	No Serious Limitations	No serious limitations	347/341	91.70 (40.52, 142.88)
GSH	No serious limitations	Very serious ^d^	No serious limitations	No serious limitations	No serious limitations	190/187	219.01 (59.30, 378.71)

^a^ The test for heterogeneity is significant, and the I^2^ is moderate, 64.0%. ^b^ The test for heterogeneity is significant, and the I^2^ is moderate, 85.2%. ^c^ The test for heterogeneity is significant, and the I^2^ is moderate, 82.2%.^d^ The test for heterogeneity is significant, and the I^2^ is moderate, 92.7% ^e^ values are distributed within opposite direction across studies.

## Data Availability

Data sharing is available.
